# Promotion of healthy adipose tissue remodeling ameliorates muscle inflammation in a mouse model of sarcopenic obesity

**DOI:** 10.3389/fnut.2023.1065617

**Published:** 2023-02-17

**Authors:** Yunlin Ge, Siqi Li, Tao Yao, Yuexiao Tang, Qiangyou Wan, Xiaoli Zhang, Jing Zhao, Mingliang Zhang, Mengle Shao, Lijun Wang, Ying Wu

**Affiliations:** ^1^The Third Department of Orthopedics, The 903th Hospital of People’s Liberation Army, Hangzhou, Zhejiang, China; ^2^CAS Key Laboratory of Molecular Virology and Immunology, The Center for Microbes, Development, and Health, Institut Pasteur of Shanghai, Chinese Academy of Sciences, Shanghai, China; ^3^University of Chinese Academy of Sciences, Beijing, China; ^4^College of Life Sciences, Zhejiang Chinese Medical University, Hangzhou, Zhejiang, China; ^5^Cancer Institute of Integrated Traditional Chinese and Western Medicine, Zhejiang Academy of Traditional Chinese Medicine, Tongde Hospital of Zhejiang Province, Hangzhou, Zhejiang, China; ^6^Key Laboratory of Cancer Prevention and Therapy Combining Traditional Chinese and Western Medicine of Zhejiang Province, Hangzhou, Zhejiang, China; ^7^Technology Service Center, Instrumental Analysis Platform, Institut Pasteur of Shanghai, Chinese Academy of Sciences, Shanghai, China; ^8^Shanghai Key Laboratory of Diabetes Mellitus, Department of Endocrinology and Metabolism, Shanghai Jiao Tong University School of Medicine Affiliated Sixth People’s Hospital, Shanghai Clinical Center for Diabetes, Shanghai Diabetes Institute, Shanghai, China; ^9^Geriatric Medicine Center, Department of Endocrinology, Zhejiang Provincial People’s Hospital, Affiliated People’s Hospital, Hangzhou Medical College, Hangzhou, Zhejiang, China; ^10^Key Laboratory of Endocrine Gland Diseases of Zhejiang Province, Affiliated People’s Hospital, Hangzhou Medical College, Hangzhou, Zhejiang, China

**Keywords:** sarcopenic obesity, adipose tissue remodeling, muscle inflammation, HIF1α, adiponectin

## Abstract

A large subset of elders is classified as having sarcopenic obesity, a prevalence of obesity in combination with sarcopenia which places an aging population at the risk of adverse health consequences from both conditions. However, its complex etiology has restrained the development of effective therapeutic strategies. Recent progress has highlighted that the mode by which adipose tissue (AT) remodels is a determinant of metabolic health in the context of obesity. Healthy AT remodeling confers metabolic protection including insulin-sensitizing and anti-inflammatory effects to non-adipose tissues including skeletal muscle. Here, we employed a doxycycline-inducible adipocyte *Hif1a* knockout system to evaluate the muscle-protective effects associated with HIF1α inactivation-induced healthy AT remodeling in a model of sarcopenic obesity. We found that adipocyte HIF1α inactivation leads to improved AT metabolic health, reduced serum levels of lipids and pro-inflammatory cytokines, and increase of circulating adipokine (APN) in ovariectomized obese mice fed with obesogenic high-fat diet (HFD). Concomitantly, muscle inflammation is evidently lower in obese OVX mice when adipocyte HIF1α is inactivated. Furthermore, these protective effects against muscle inflammation can be mimicked by the administration of adiponectin receptor agonist AdipoRon. Collectively, our findings underscore the importance of AT metabolic health in the context of concurrent sarcopenia and obesity, and promotion of healthy AT remodeling may represent a new therapeutic strategy to improve muscle health in sarcopenic obesity.

## Introduction

Obesity is a chronic metabolic disorder which significantly increases the risk of many human diseases including diabetes, cardiovascular disease, and certain types of cancers (1, 2). Within the fast-aging global population, the steady increase of obesity rates is estimated to offset gains in life expectancy worldwide in the coming decades (3, 4). Sarcopenia, another naturally occurring disorder in elderly population, is defined as a progressive skeletal muscle disorder that involves the loss of muscle mass and strength or physical function (5). In older people, sarcopenia is clinically associated with increased adverse outcomes like falls, frailty and functional decline and mortality (6). As the confluence of these two age-related conditions, sarcopenic obesity is characterized as co-occurrence of sarcopenia and obesity whose synergistic action profoundly affects metabolic organs and systemic metabolic health. A growing body of evidence shows that sarcopenic obesity has greater effects on the development of age-related metabolic diseases and mortality than either sarcopenia or obesity alone (7).

With respect to the etiology of sarcopenic obesity, multiple regulatory factors and pathways are involved in the regulation of body composition during aging. In humans, the decline of muscle mass begins in the fourth decade of life, coinciding with a steady increase of fat mass (8, 9). These age-dependent changes are largely due to the decrease in the components of total energy expenditure (physical activity, adaptive thermogenesis, etc.), accompanied with altered circulating levels of numerous circulating hormones which are active regulators of muscle and fat metabolism. Of note, sex hormone-regulated changes in muscle and fat composition represent an essential part of age-dependent metabolic conditions associated with sarcopenic obesity. In women, menopause increases body weight and visceral fat mass, but decreases muscle mass, highlighting the regulatory role of estrogen on body composition (10, 11). Estrogen deficiency-induced shift of body composition is recapitulated in young female rodents following surgical ovary removal, or “ovariectomy,” therefore ovariectomized mice are used in studies as a model of concurrent muscle wasting and fat accumulation (12–14).

Inflammation is a common feature in both sarcopenia and obesity. In aging and obesity, activation of inflammatory pathways leads to insulin resistance and decreased muscle catabolism, driving the loss of muscle mass (8). It is appreciated that AT is an active endocrine organ secreting numerous bioactive adipokines (such as leptin and adiponectin) and pro-inflammatory cytokines (such as TNFα and IL-6) (15, 16). On the other hand, the levels of adiponectin (APN), an insulin-sensitizing and anti-inflammatory adipokine, are inversely correlated with body weight and age (17, 18), implying the involvement of adipokines in the regulation of muscle mass loss in the setting of sarcopenic obesity.

It is increasingly clear that the metabolic health of AT is not determined by adiposity *per se*, but the manner in which AT expands and remodels represents a crucial indicator of developing metabolic disorders related to AT dysfunction (19, 20). Pathological AT remodeling, typically featured by adipocyte hypertrophy, chronic inflammation, hypoxia, and fibrosis, is generally associated with hyperlipidemia and “lipid spillover” to non-adipose organs including liver and muscle. This ectopic lipid deposition leads to local inflammation, insulin resistance and even tissue damage (21). AT is featured by its extraordinary plasticity in response to external signals, and the mode by which it remodels is controlled by a series of regulatory factors and pathways. Dysfunctional AT usually exhibits a higher degree of hypoxia response, and the consequential activation of hypoxia-inducible factor1α (HIF1α) signaling pathway drives AT fibrosis, limits *de novo* adipogenesis and promotes adipocyte hypertrophy (21). Healthy AT remodeling, observed in “metabolically healthy” obese individuals, is generally associated with improvements in angiogenesis and AT hypoxia, which alleviates pathological activation of HIF1α (21). In mouse models, suppression of HIF1α activity in mature adipocytes and adipocyte progenitors protects AT from pathologic remodeling and ameliorates metabolic disorders in diet-induced obesity (22–25).

Here, we utilized a doxycycline-inducible adipocyte *Hif1a* knockout mice to evaluate the muscle-protective effects of metabolically healthy AT remodeling in the context of co-occurring sarcopenia and obesity. Our results reveal that pharmacological and genetic HIF1α inhibition improves AT metabolic homeostasis, accompanied by decreased circulating levels of lipids and pro-inflammatory cytokines, but increased metabolically beneficial adiponectin in ovariectomized obese mice fed with obesogenic diet. These remarkable metabolic improvements are associated with improved muscle inflammation in this mouse model of sarcopenic obesity. Moreover, the protective effect on muscle inflammation is mimicked by the administration of pharmacological adiponectin receptor agonist, underlying the possibility of therapeutically targeting AT as a means to prevent muscle damage and preserve muscle health in sarcopenic obesity.

## Materials and methods

### Animals

*Adipoq*^rtTA^[C57BL/6-Tg(Adipoq-rtTA)2Zvw/J, JAX#:033448], and *TRE-Cre*[B6.Cg-Tg(tetO-cre)1Jaw/J, JAX#:006234] strains were purchased from Jackson laboratory. *Hif1a*^loxP/loxP^ mice were generously provided by Dr. Liwei Xie, Guangdong Academy of Sciences. Mice were maintained in ventilated cages under an inverted 12-h:12-h dark:light cycle with *ad libitum* access to food and water. All wild-type (WT) and transgenic animals used in this study were on the C57BL/6 background.

### Rodent diets and drug treatments

Transgene expression was induced with a standard rodent chow diet or chow diet containing 600 mg/kg doxycycline (DOX) (Bio-Serv, S4107). For high-fat diet studies, mice were maintained on a standard high-fat diet (60 kcal% fat, Research Diets, D12492i) or doxycycline-containing high fat diet (600 mg/kg dox, 60% kcal% fat, Bio-Serv, S5867) as indicated. For PX-478 administration, mice were administered intraperitoneally with saline or 5 mg/kg of PX-478 (Cayman Chemical, #10005189) three times weekly for 4 weeks. For AdipoRon administration, mice received intraperitoneal injection with saline or 5 mg/kg of AdipoRon (Cayman Chemical, #15941) twice a week.

### Body composition analysis

The Bruker Minispec mq10 NMR were used to determine the body fat mass and lean mass in conscious mice.

### Tissue and serum measurements

Triglyceride determination kit (Sigma, triglyceride reagent T2449 and free glycerol reagent F6428) were used to measure the serum levels of triglycerides of the mice. Serum adiponectin concentrations were determined using ELISA kit (Sigma-Aldrich, EZMADP-60K). Tissue TNFα and IL6 levels were measured using ELISA kits (BioLegend, 430901; Sigma-Aldrich, RAB0309).

### Immunoblotting and antibodies

Protein extracts from adipose tissue were prepared by homogenization in NP-40 lysis buffer (Beyotime, P0013F) supplemented with Protease Inhibitor Cocktail (Sigma, P8340). Protein extracts were separated by SDS-PAGE electrophoresis and transferred onto PVDF membrane (Millipore, IPFL00010). After incubation with the indicated primary antibodies at 4°C overnight, the blots were incubated with secondary antibodies and visualized by Pierce ECL Plus Western Blotting Substrate (Thermo Fisher Scientific, 32109).

The primary antibodies and the working concentrations are as following:

HIF1α: 1:1000 dilution; Cell Signaling Technology, 36169.

β-TUBULIN: 1:1000 dilution; Cell Signaling Technology, 2128.

### Gene expression analysis

Total RNA was isolated from freshly isolated tissue using the TRIzol reagent (Invitrogen, 15596018). First strand cDNA was reverse-transcribed with M-MLV reverse transcriptase (Invitrogen, 28025-021) and random hexamer primers (Invitrogen, 48190011). Relative expression of mRNAs was determined by quantitative PCR using a TransStart^®^ Tip Green qPCR SuperMix (TransGen Biotech, AQ132-21) and data was analyzed using the DD-Ct method of gene quantification with *Rps18* acting as the reference transcript for mRNA. All qPCR primer sequences are listed as following: *Tnfa* (Forward 5′-GAAAGGGGATTATGGCTCAGG-3′) (Reverse 5′-TCACTGTCCCAGCATCTTGTG-3′), *Il6* (Forward 5′-AAGCCAGA GTCCTTCAGAGAGA-3′) (Reverse 5′-ACTCCTTCTGTGACTCC AGCTT-3′), *Saa3* (Forward 5′-TGCCATCATTCTTTGCATCTT GA-3′) (Reverse 5′-CCGTGAACTTCTGAACAGCCT-3′), *Ccl2* (Forward 5′-CCACAACCACCTCAAGCACTTC-3′) (Reverse 5′-AA GGCATCACAGTCCGAGTCAC-3′), *Fn1* (Forward 5′-GAGAGCA CACCCGTTTTCATC-3′) (Reverse 5′-GGGTCCACATGATGGTG ACTT-3′), *Lox* (Forward 5′-TCGCTACACAGGACATCATGC-3′) (Reverse 5′-ATGTCCAAACACCAGGTACGG-3′), *Adgre1* (Forward 5′-TTGTACGTGCAACTCAGGACT-3′) (Reverse 5′-GATCCCAGA GTGTTGATGCAA-3′), *Itgam* (Forward 5′-ATGGACGCTGATG GCAATACC-3′) (Reverse 5′-TCCCCATTCACGTCTCCCA-3′), *Il1b* (Forward 5′-GCAACTGTTCCTGAACTCAACT-3′) (Reverse 5′-ATCTTTTGGGGTCCGTCAACT-3′), and *Rps18* (Forward 5′-CATGCAAACCCACGACAGTA-3′) (Reverse 5′-CCTCACGC AGCTTGTTGTCTA-3′).

### Histological analysis

Tissues were dissected in PBS (Beyotime, ST448) and fixed in 4% paraformaldehyde (Beyotime, P0099) overnight. Paraffin embedding, sectioning, and H&E staining, were performed by the Wuhan Servicebio Technology Co., Ltd. The following antibodies and concentrations were used for indirect immunofluorescence assays: guinea pig anti-perilipin 1:500 (Fitzgerald 20R-PP004); mouse anti-F4/80 1:500 (Cell Signaling Technology, 70076S); goat anti-rabbit Alexa 488 1:200 (Invitrogen, A-11008); goat anti-guinea pig Alexa 647 1:200 (Invitrogen, A21450). Briefly, after dewaxing and rehydration, antigen retrieval of the sections was performed by microwave oven with buffer of citric acid (Beyotime, P0086) for 14 min. Afterward, sections were washed in water and PBS for 5 min, three times and then blocked with 10% normal goat serum (Sigma-Aldrich, G9023) in PBS for at least 30 min at room temperature. Primary antibodies were diluted in PBS containing 10% normal goat serum and sections were incubated with the primary antibodies overnight at 4°C. Following three times of wash with PBS, sections were incubated with secondary antibodies diluted in PBS containing 10% normal goat serum for 2 h at room temperature. Nuclei were counterstained with ProLong™ Gold Antifade Mountant with DAPI (Invitrogen, P36931). Bright-field and fluorescent images were acquired using ECHO RVL-100-G microscope. Confocal micrographs were captured using Olympus SpinSR10 Ixplore system at the Live Cell Imaging Core at Institut Pasteur of Shanghai.

### Statistical analysis

All data were expressed as the mean ± SEM. Statistical analyses were performed using GraphPad Prism7.0 (GraphPad Software, Inc., La Jolla, CA, USA). A two-tailed unpaired Student’s *t*-test was performed to assess the statistical significance between two independent groups. A *p* value of 0.05 was set as the significance threshold. The predicted numbers of independent replicates per group were estimated based on independent preliminary studies and experience.

## Results

### High fat diet exacerbates muscle inflammation in ovariectomized mice

**FIGURE 1:**
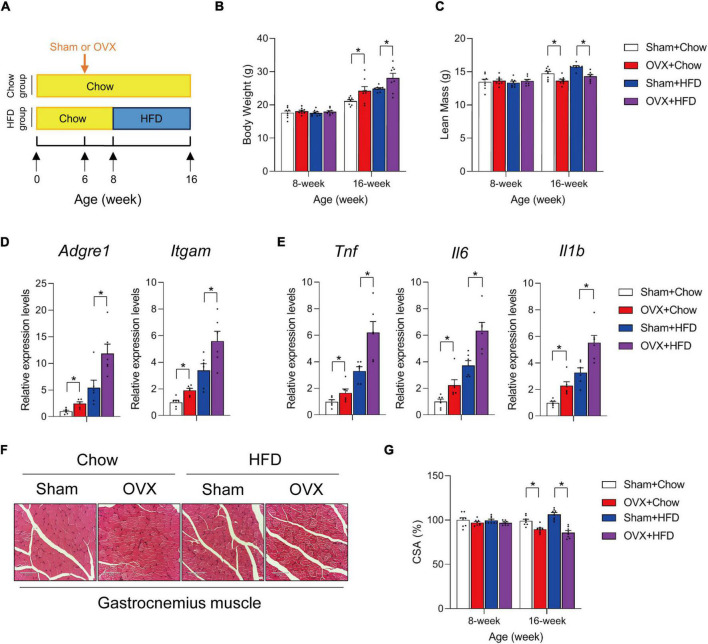
High fat diet exacerbates muscle inflammation in ovariectomized mice. **(A)** Schematic diagram illustrating experimental design. Six-week old wildtype C57BL/6 female mice kept on standard chow diet were operated (sham or ovariectomy/OVX). Two weeks after the operation, sham and ovariectomized (OVX) mice were divided into chow- or high-fat diet (HFD)-feeding groups and kept on the diet regimen for another 8 weeks. **(B)** Body weights of each group were measured before and after HFD feeding. *n* = 8 per group. Bars represent mean ± SEM. *p < 0.05 by two-way ANOVA. **(C)** Lean mass of each group after 8 weeks of HFD feeding. *n* = 8 per group. Bars represent mean ± SEM. *p < 0.05 by two-way ANOVA. **(D)** Relative expression of macrophage marker genes in gastrocnemius muscle from mice of the indicated groups. *n* = 6 per group. Bars represent mean ± SEM. *p < 0.05 by two-way ANOVA. **(E)** Relative expression of inflammation-related genes in gastrocnemius muscle from mice of the indicated groups. *n* = 6 per group. Bars represent mean ± SEM. *p < 0.05 by two-way ANOVA. **(F)** Representative H&E staining of gastrocnemius muscle sections from mice of the indicated groups. Scale bar = 100 μm. **(G)** Cross-sectional area (CSA) of gastrocnemius muscle from mice of the indicated groups. *n* = 8. Bars represent mean ± SEM. *p < 0.05 by two-way ANOVA.

To evaluate the effects of HFD on muscle health in the setting of sarcopenic obesity, standard chow diet-fed 6-week old C57BL/6 female mice were ovariectomized (OVX) or sham-operated. Two weeks after the operations, mice were divided into four groups: sham + chow, sham + HFD, OVX + chow, and OVX + HFD, and the animals were kept on indicated diets for another 8 weeks (Figure 1A). As expected, OVX and HFD significantly increased body weight on female mice (Figure 1B), and HFD feeding led to additional body weight gain of OVX mice (Figure 1B). Of note, 10 weeks after the operations, OVX led to ∼10% loss of lean mass in female mice, while HFD did not show additional effects on muscle wasting in OVX mice (Figure 1C). H&E staining of gastrocnemius muscle sections confirmed the reduction in cross-sectional area (CSA) in OVX mice, but did not indicate that HFD caused apparent morphological difference compared to chow-fed mice subjected to the same operations (Figures 1F, G). However, despite of the minimal impact of 8-week HFD feeding on muscle wasting in OVX mice, qPCR measurements of macrophage marker genes and expression of pro-inflammatory cytokine genes in gastrocnemius muscle revealed heightened immune cell activation and inflammation levels in HFD-fed OVX mice (Figures 1D, E). Hence, HFD *per se* did not exacerbate muscle wasting in OVX sarcopenic obesity model, but the muscle inflammation was markedly augmented in OVX fed with HFD.

### Adipocyte HIF1α activation promotes AT inflammation in high fat diet-fed ovariectomized mice

Diet-induced obesity is associated with remarkable AT expansion and remodeling (1, 26). The manner by which AT remodels determines AT metabolic health and profoundly affects non-adipose organs (e.g., liver and skeletal muscle) and systemic energy homeostasis (20, 21). In obesity, pathological AT remodeling featured by increased levels of inflammation, fibrosis and adipocyte hypertrophy is driven by a variety of key regulatory pathways including HIF1α signaling pathway (22–25). Chemical inhibition of HIF1α by PX-478 is effective to improve AT metabolic health in obese animal models (24, 25). In agreement, PX-478 treatment protected HFD-fed OVX mice against pathological AT remodeling (Supplementary Figure 1). C57BL/6 female mice were kept on chow-diet till 6 weeks of age prior to sham or OVX operations. Two weeks after the operations, mice were switched to HFD feeding for another 8 weeks during which the animals were administrated by vehicle or PX-478 (Supplementary Figure 1A). PX-478 treatment did not lead to difference in body weight or lean mass between sham-operated and OVX mice (Supplementary Figures 1B, C). As expected, PX-478 treatment significantly reduced the inflammation- and fibrosis-related genes in AT isolated from obese OVX mice (Supplementary Figure 1D). Of note, PX-478 administration decreased inflammation gene expression in gastrocnemius muscle of obese OVX mice, despite of the lack of apparent difference of muscle morphology and CSA (Supplementary Figures 1E–G). These data implied a potential link between improved AT metabolic remodeling which benefits muscle health in the setting of sarcopenic obesity.

**FIGURE 2:**
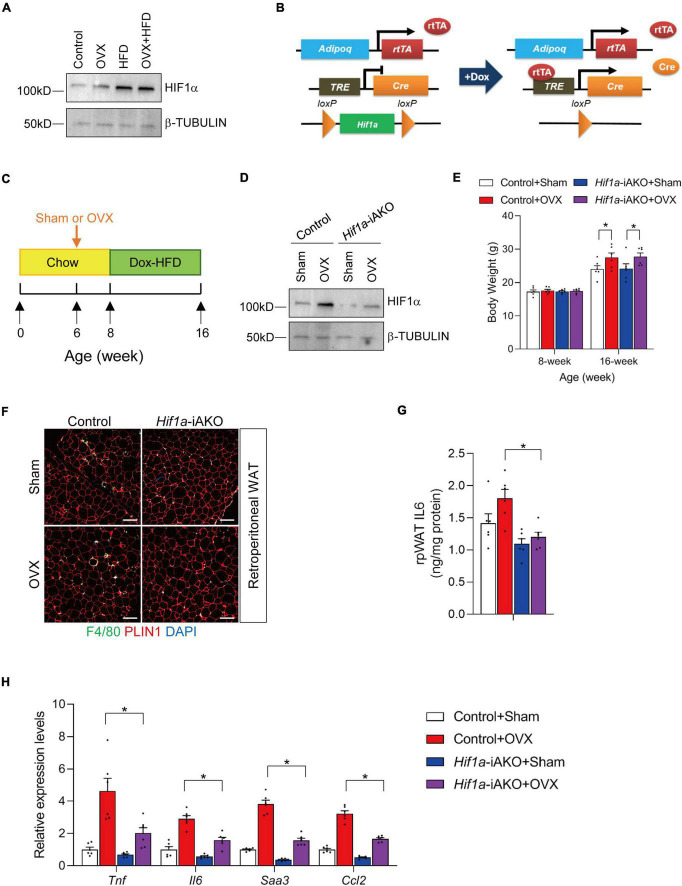
Adipocyte *Hif1a* deletion drives healthy adipose tissue (AT) expansion in high-fat diet (HFD)-fed ovariectomized mice. **(A)** Immunoblot analysis of protein levels of HIF1α in retroperitoneal AT isolated from sham or OVX mice fed with chow or high fat diet (HFD) as illustrated in Figure 1A. **(B)** Schematic illustration of *Hif1a*-iAKO mice. *Adipoq*^rtTA^; *TRE-Cre*; *Hif1a*^loxP/loxP^ (*Hif1a*-iAKO) mice are generated by breeding *Adipoq*^rtTA^ transgenic mice to animals carrying Cre recombinase under the control of tet-reponse element (*TRE-Cre*) and floxed *Hif1a* alleles (*Hif1a*^loxP/loxP^). Littermates carrying only *Adipoq*^rtTA^; *Hif1a*^loxP/loxP^ allelles (i.e., Cre^–^) were used as control animals. **(C)** Schematic diagram illustrating experimental design. Six-week old control or *Hif1a*-iAKO female mice kept on standard chow diet were operated (sham or OVX). 2 weeks after the operation, mice were switched to doxycycline (Dox)-containing HFD for another 8 weeks. **(D)** Immunoblot analysis of protein levels of HIF1α in retroperitoneal AT isolated from DOX HFD-fed control or *Hif1a*-iAKO female mice subjected to sham or OVX operations as illustrated in Figure 2B. **(E)** Body weights of each group were measured before and after HFD feeding. n = 6 per group. Bars represent mean ± SEM. *p < 0.05 by two-way ANOVA. **(F)** Representative immunofluorescence staining using anti-PLIN1 and anti-F4/80 antibodies of retroperitoneal AT (rpAT) sections from mice of the indicated groups after HFD feeding. Scale bar = 200 μm. **(G)** Levels of IL6 in rpAT of mice from the indicated groups after HFD feeding. *n* = 6 per group. **(H)** Relative of inflammation-related genes in rpAT from mice of the indicated groups after HFD feeding. *n* = 6 per group. Bars represent mean ± SEM. *p < 0.05 by two-way ANOVA.

To determine that healthy AT remodeling improves muscle inflammation in HFD-fed OVX mice, we employed an adipocyte-specific HIF1α loss-of-function genetic model. Mature adipocyte HIF1α activation is a potent regulator of fibrogenic and pro-inflammatory gene program (22, 24). Genetically modified mice in which *Hif1a* is constitutively inactivated in *Adipoq*-expressing mature adipocytes are protected from unhealthy AT remodeling in diet-induced obesity (22, 23). First, we detected slightly increased HIF1α protein levels in retroperitoneal AT isolated from OVX mice, and greater increase of HIF1α abundance was observed in HFD-fed sham and OVX female mice (Figure 2A). To assess whether improved AT metabolic health caused by adipocyte HIF1α inactivation would have beneficial effects on muscle health in the setting of sarcopenic obesity, we generated a mouse model in which adipocyte *Hif1a* was inactivated in a doxycycline (DOX)-inducible manner (Figure 2B). This genetic mouse model consists of a transgene that expresses the reverse tetracycline transactivator (rtTA) under the control of a 5.4 kb promoter sequence from *adipoq* locus, another transgene in which Cre recombinase is expressed from a promoter containing Tet-response element (TRE-Cre), and two “floxed” (loxP-flanked) *Hif1a* alleles (Adipoq^rtTA^; TRE-Cre; *Hif1a*^loxP/loxP^ mice, herein denoted as *Hif1a*-iAKO mice) (Figure 2B). Female *Hif1a*-iAKO mice and littermate controls (Adipoq^rtTA^; *Hif1a*^loxP/loxP^ mice) were kept on chow diet until 6 weeks of age prior to OVX or sham operations. 2 weeks after the operations, mice were switched to DOX (600 mg/kg)-containing HFD diet for another 8 weeks (Figure 2C). Immunoblot analysis was used to determine the inactivation of HIF1α in retroperitoneal AT in *Hif1a*-iAKO mice following the DOX treatment (Figure 2D). Control and *Hif1a*-iAKO mice subjected to OVX gained extra body weight to a comparable extend after DOX-HFD feeding (Figure 2E). Immunofluorescence staining of AT sections isolated from *Hif1a*-iAKO mice showed reduction of crown-like structure (CLS) accumulation compared to control mice after HFD-feeding, and *Hif1a* deletion in adipocytes induced the decreased levels of IL6 in retroperitoneal AT, which are both indicative of improved AT immune cell infiltration and inflammation (Figures 2F, G). Indeed, the expression levels of inflammation-related genes (*Tnf*, *Il6*, *Saa3*, and *Ccl2*) were significantly decreased in ovariectomized *Hif1a*-iAKO mice after HFD-feeding (Figure 2H). These data jointly proved that adipocyte *Hif1a* inactivation led to improved AT inflammation in OVX mice fed with HFD.

### Promotion of AT metabolic health is associated with ameliorated muscle inflammation

**FIGURE 3:**
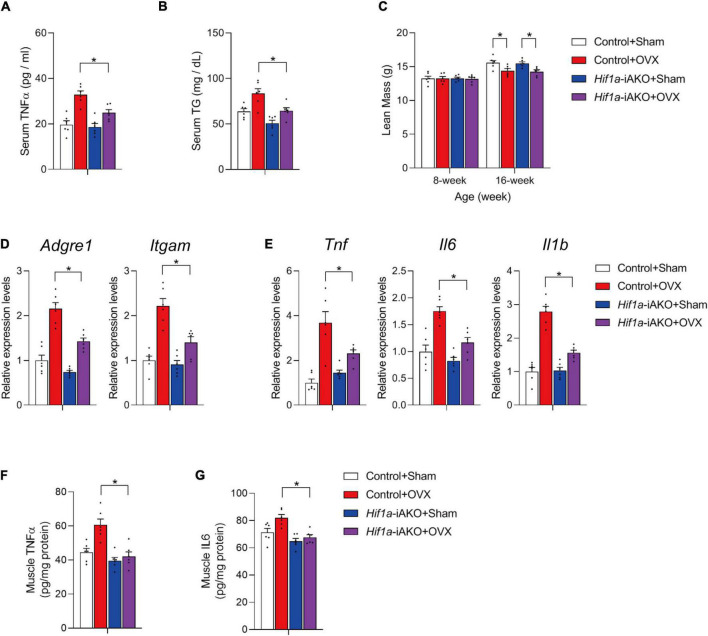
Improved muscle inflammation in high-fat diet (HFD)-fed ovariectomized *Hif1a*-iAKO mice. **(A)** Serum TNFα levels in mice of the indicated groups after HFD feeding. *n* = 6 per group. Bars represent mean ± SEM. *p < 0.05 by two-way ANOVA. **(B)** Serum levels of triglycerides in mice of the indicated groups after HFD feeding. *n* = 6 per group. Bars represent mean ± SEM. *p < 0.05 by two-way ANOVA. **(C)** Lean mass of the indicated groups. *n* = 6. Bars represent mean ± SEM. *p < 0.05 by two-way ANOVA. **(D)** Relative expression of macrophage marker genes in gastrocnemius muscle from mice of the indicated groups. *n* = 6 per group. Bars represent mean ± SEM. *p < 0.05 by two-way ANOVA. **(E)** Relative expression of inflammation-related genes in gastrocnemius muscle from mice of the indicated groups. *n* = 6 per group. Bars represent mean ± SEM. *p < 0.05 by two-way ANOVA. **(F)** Levels of TNFα in muscle of mice from the indicated groups after HFD feeding. *n* = 6 per group. **(G)** Levels of IL6 in muscle of mice from the indicated groups after HFD feeding. *n* = 6 per group.

Many genetic models have provided evidence that healthy AT remodeling, independent of decrease in adiposity, benefits non-adipose metabolic organs in the contexts of limited release of deleterious pro-inflammatory factors from dysfunctional AT, and the reduction of circulating lipid levels (27–34). In OVX *Hif1a*-iAKO mice, the serum levels of TNFα and triglycerides (TG) were significantly lower compared to those in control mice after HFD feeding (Figures 3A, B). These metabolic benefits did not improve lean mass loss in HFD-fed OVX *Hif1a*-iAKO mice (Figure 3C), but led to marked reduction of muscle inflammation reflected by the decreased expression of macrophage markers and inflammation-related genes in gastrocnemius muscle isolated from ovariectomized control and *Hif1a*-iAKO mice after HFD-feeding (Figures 3D, E). In alignment, the tissue levels of pro-inflammatory cytokine TNFα and IL6 were also significantly suppressed in muscle samples from ovariectomized control and *Hif1a*-iAKO mice after HFD-feeding (Figures 3F, G). Of note, these effects were largely absent in sham-operated *Hif1a*-iAKO mice compared with their control counterparts (Figures 3D–G), implying that the protective effects produced by improved AT metabolic remodeling on muscle inflammation are selectively more robust in the setting of greater HIF1α activation (Figure 2A).

### Adiponectin receptor agonist improves muscle inflammation in ovariectomized mice fed with high fat diet

**FIGURE 4:**
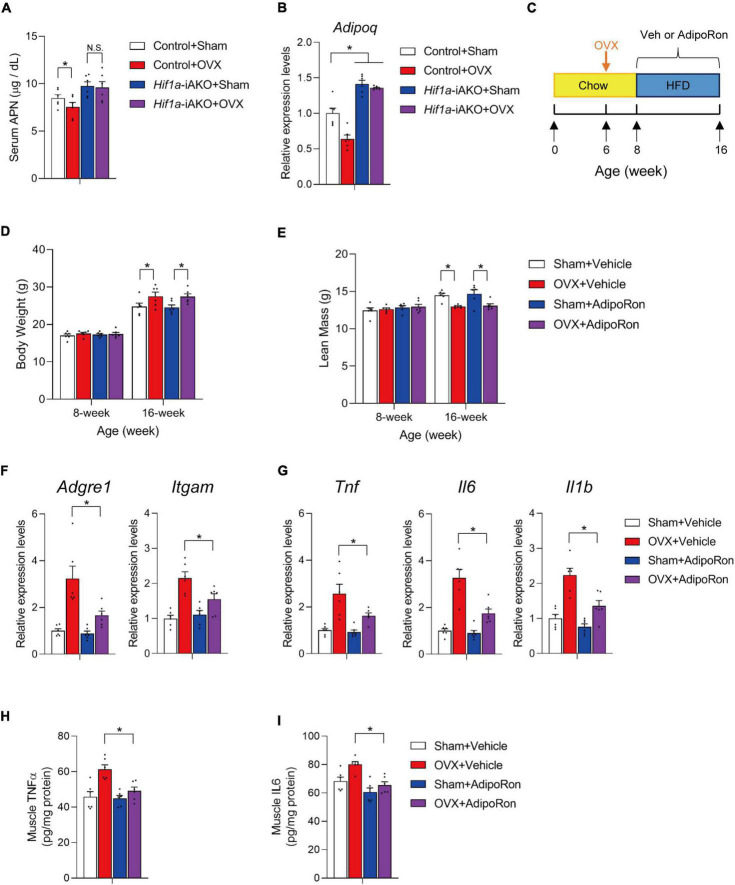
AdiopRon administration reduces muscle inflammation in high-fat diet (HFD)-fed ovariectomized mice. **(A)** Serum Adiponectin (APN) levels in mice of the indicated groups after HFD feeding. *n* = 6 per group. Bars represent mean ± SEM. *p < 0.05 by two-way ANOVA. **(B)** Relative expression of *Adipoq* in fractionated retroperitoneal AT adipocytes of the indicated groups after HFD feeding. *n* = 6 per group. Bars represent mean ± SEM. *p < 0.05 by two-way ANOVA. **(C)** Schematic diagram illustrating experimental design. Six-week old wildtype C57BL/6 female mice kept on standard chow diet were operated (sham or OVX). Two weeks after the operation, sham and ovariectomized (OVX) mice were switched to HFD feeding with vehicle or AdipoRon treatment for another 8 weeks. **(D)** Body weight of each group were measured before and after HFD feeding. *n* = 6 per group. Bars represent mean ± SEM. *p < 0.05 by two-way ANOVA. **(E)** Lean mass of each group after 8 weeks of HFD feeding. *n* = 6 per group. Bars represent mean ± SEM. *p < 0.05 by two-way ANOVA. **(F)** Relative expression of macrophage marker genes in gastrocnemius muscle from mice of the indicated groups. n = 6 per group. Bars represent mean ± SEM. *p < 0.05 by two-way ANOVA. **(G)** Relative expression of inflammation-related genes in gastrocnemius muscle from mice of the indicated groups. *n* = 6 per group. Bars represent mean ± SEM. *p < 0.05 by two-way ANOVA. **(H)** Levels of TNFα in muscle of mice from the indicated groups after HFD feeding. *n* = 6 per group. **(I)** Levels of IL6 in muscle of mice from the indicated groups after HFD feeding. *n* = 6 per group.

In addition to its effects on AT production of deleterious factors, improved AT remodeling also leads to elevated levels of metabolically beneficial factors (2, 15). The best example is the increased levels of adiponectin (APN), an anti-inflammatory and insulin-sensitizing adipokine, in genetic models with improved AT metabolic health (17, 27, 31), and in metabolically healthy obese individuals whose AT exhibits features of healthy remodeling (35–37). In agreement, our OVX *Hif1a*-iAKO mice preserved similar serum APN levels compared to those in sham control mice (Figure 4A), and mRNA level of *Adipoq* in fractionated adipocytes were upregulated in both *Hif1a*-iAKO groups (Figure 4B), which prompted us to speculate that the increased circulating APN could contribute to the protection against muscle inflammation and APN chemical agonist could be effective in alleviating muscle conditions in this setting of sarcopenic obesity. Therefore, we next set to determine if the administration of adiponectin receptor agonist AdipoRon (38) is effective to recapitulate, at least in part, the protective effects on skeletal muscle in obese OVX mice. Therefore, a cohort of C57BL/6 female mice were kept on chow-diet till 6 weeks of age prior to sham or OVX operations. Two weeks after the operations, mice were switched to HFD feeding for another 8 weeks during which the animals were administrated by vehicle or AdipoRon (5 mg/kg) twice a week (Figure 4C). During this course, the administration of AdipoRon did not show obvious effects on body weight gain or lean mass loss in OVX mice (Figures 4D, E). But, the qPCR analysis of macrophage marker and inflammation-related genes in gastrocnemius muscle showed that AdipoRon administration was effective to ameliorate muscle inflammation in OVX mice after HFD feeding (Figures 4F, G), which is associated with reduced levels of pro-inflammatory cytokines TNFα and IL6 in muscle samples isolated from AdipoRon treated OVX mice after HFD feeding (Figures 4H, I). Hence, pharmacological adiponectin receptor activation effectively improved muscle inflammation in OVX mice fed with HFD.

## Discussion

The prevalence of sarcopenic obesity owing to the rapidly aging population and obesity pandemic has placed a growing number of people at the risk of related health consequences including metabolic diseases, disability and mortality (7, 8).

Sex hormones are important regulators of muscle mass, fat mass and body composition (10, 11, 39, 40), and age-dependent changes of estrogen and androgen levels are suggested to be responsible for the pathology of sarcopenic obesity (8, 12). Testosterone promotes muscle regeneration *via* its effects on satellite cells and muscle protein synthesis, and thus is considered as protective in preserving muscle mass and strengthen in aged male individuals (39, 40). In postmenopausal women, the lack of estrogen causes sex-specific body composition changes of increased visceral fat mass and decreased muscle mass (10, 41). In rodents, estrogen deficiency caused by surgical ovariectomy leads to compromised recovery of muscle atrophy (42), muscle contractile dysfunction (43), and progressive loss of muscle mass and strength (14), associated with the steady increase of fat mass (13, 14). Therefore, OVX mice represent an experimental model which recapitulates defining characteristics of sarcopenic obesity resulted from sex hormone changes.

In addition to hormonal mechanism, activation of inflammatory pathways, a hallmark of obesity, is indicated in the progression of muscle wasting and dysfunction (8, 12). Obesity results in the elevated circulating levels of pro-inflammatory cytokines such as TNFα which can directly affect muscle protein synthesis and mitochondrial activity (44). Besides, obesity-associated chronic inflammation leads to the development of peripheral insulin resistance and increased muscle catabolism (45, 46). Meanwhile, anti-inflammatory adiponectin is inversely correlated with obesity and age, a mechanism considered to exacerbate inflammation in peripheral tissues including skeletal muscle (17, 47, 48).

Recent research progress has revealed that promotion of healthy AT remodeling is metabolically protective in obesity, owing to the alleviation of obesity-associated insulin resistance and inflammation (1, 20, 26). Here, we provide direct evidence that improved AT metabolic health is sufficient to exert protection to skeletal muscle in the setting of sarcopenic obesity. Using the adipocyte-specific *Hif1a* knockout mice, we were able to promote healthy AT expansion in HFD-fed OVX mice. These metabolic improvements in AT led to lower circulating levels of TNFα and TG, which is associated with reduced inflammation in skeletal muscle in OVX *Hif1a*-iAKO mice fed with HFD. In addition, we also evaluated the beneficial effects of adiponectin receptor agonist on muscle health in HFD-fed OVX mice. Low APN levels are known to be related to progression of a number of pathological complications such as insulin resistance, nephropathy, retinopathy, and cardiomyopathy (17, 49). There is convincing evidence of the relationship between APN and muscle development, growth and maintenance (47), largely due to its regulation of inflammatory response and insulin sensitivity in peripheral tissues (50–52). Circulating APN levels are reduced in obesity and represent a sensitive indicator of AT metabolic health (17, 53). Healthy AT remodeling is associated with heightened levels of APN, and our data from *Hif1a*-iAKO mice confirmed that healthy AT remodeling increases APN levels in HFD-fed OVX mice, suggestive of the contribution of this anti-inflammatory adipokine to regulate muscle inflammation. Indeed, pharmacological activation of APN receptor by chemical agonist AdipoRon markedly decreases muscle inflammation in HFD-fed OVX mice. Collectively, our findings support that improved AT metabolic health reduce the production of deleterious factors and increases blood concentration of beneficial APN to coordinately ameliorate muscle inflammation in HFD-fed OVX mice. Of note, in alignment with the more robust activation of HIF1α in AT in the setting of HFD feeding, most effects resulted from adipocyte *Hif1a* inactivation were not as significant when mice were only subjected to OVX or HFD alone, suggesting that AT metabolic health is greater in importance in the situation of HFD-exacerbated sarcopenic obesity. Lastly, our data provide proof-of-concept evidence that pharmacological mimetics of adipose tissue-derived beneficial factors is a possible strategy to improve muscle health in the context of sarcopenic obesity.

## Conclusion

In summary, OVX mice fed with HFD exhibited both apparent features of both sarcopenia and obesity, which enables the evaluation of the protective effects caused by metabolically healthy AT remodeling. Hence, we generated a doxycycline-inducible adipocyte *Hif1a* knockout mice with features of metabolically healthy AT remodeling in the context of concurrent sarcopenia and obesity. We demonstrated that pharmacological and genetic inhibition of HIF1α can promote healthy AT metabolic remodeling in HFD-fed OVX mice, which confers protection against muscle inflammation. These benefits were associated with increased levels of serum APN, an adipokine with anti-inflammatory properties. Administration of adiponectin receptor agonist led to reduced muscle inflammation of HFD-fed OVX mice, indicating that adipokines were in part responsible for the muscle-protective benefits caused by enhanced healthy AT remodeling. Collectively, we report a proof-of-concept study that promotion of healthy AT remodeling is effective to alleviate muscle inflammation associated with sarcopenic obesity. The anti-inflammatory effects of metabolically protective adipokines may represent new therapeutic opportunities for symptom palliation in sarcopenic obesity.

## Data availability statement

The original contributions presented in this study are included in this article/Supplementary material, further inquiries can be directed to the corresponding authors.

## Ethics statement

The animal study was reviewed and approved by Zhejiang Academy of Traditional Chinese Medicine.

## Author contributions

MS, LW, and YW designed the study and wrote the manuscript. YG, SL, TY, and YT designed and executed the experiments. QW, XZ, JZ, and MZ provided critical reagents and technical support. All authors performed data analysis and approved the submitted version.

## Supplementary material

Supplementary Figure 1 HIF1α inhibitor improves adipose tissue remodeling and muscle inflammation in high-fat diet (HFD)-fed ovariectomized mice. **(A)** Schematic diagram illustrating experimental design. Six-week old wildtype C57BL/6 female mice kept on standard chow diet were operated (sham or OVX). Two weeks after the operation, sham and ovariectomized (OVX) mice were switched to HFD feeding with vehicle or PX-478 treatment for another 8 weeks. **(B)** Body weights of each group were measured before and after HFD feeding. *n* = 6 per group. Bars represent mean ± SEM. **p* < 0.05 by two-way ANOVA. **(C)** Lean mass of each group after 8 weeks of HFD feeding. *n* = 6 per group. Bars represent mean ± SEM. **p* < 0.05 by two-way ANOVA. **(D)** Relative of inflammation-related genes in rpAT from mice of the indicated groups after HFD feeding. *n* = 6 per group. Bars represent mean ± SEM. **p* < 0.05 by two-way ANOVA. **(E)** Representative H&E staining of gastrocnemius muscle sections from mice of the indicated groups. Scale bar = 100 μm. **(F)** Cross-sectional area (CSA) of gastrocnemius muscle from mice of the indicated groups. *n* = 6 per group. Bars represent mean ± SEM. **p* < 0.05 by two-way ANOVA. **(G)** Relative expression of inflammation-related genes in gastrocnemius muscle from mice of the indicated groups. *n* = 6 per group. Bars represent mean ± SEM. **p* < 0.05 by two-way ANOVA.
