# Supplementary figures and images for: Promotion of healthy adipose tissue remodeling ameliorates muscle inflammation in a mouse model of sarcopenic obesity
Source: Front Nutr. 2023 Feb 17;10:1065617. doi: 10.3389/fnut.2023.1065617 (PMC9982752; doi:10.3389/fnut.2023.1065617)

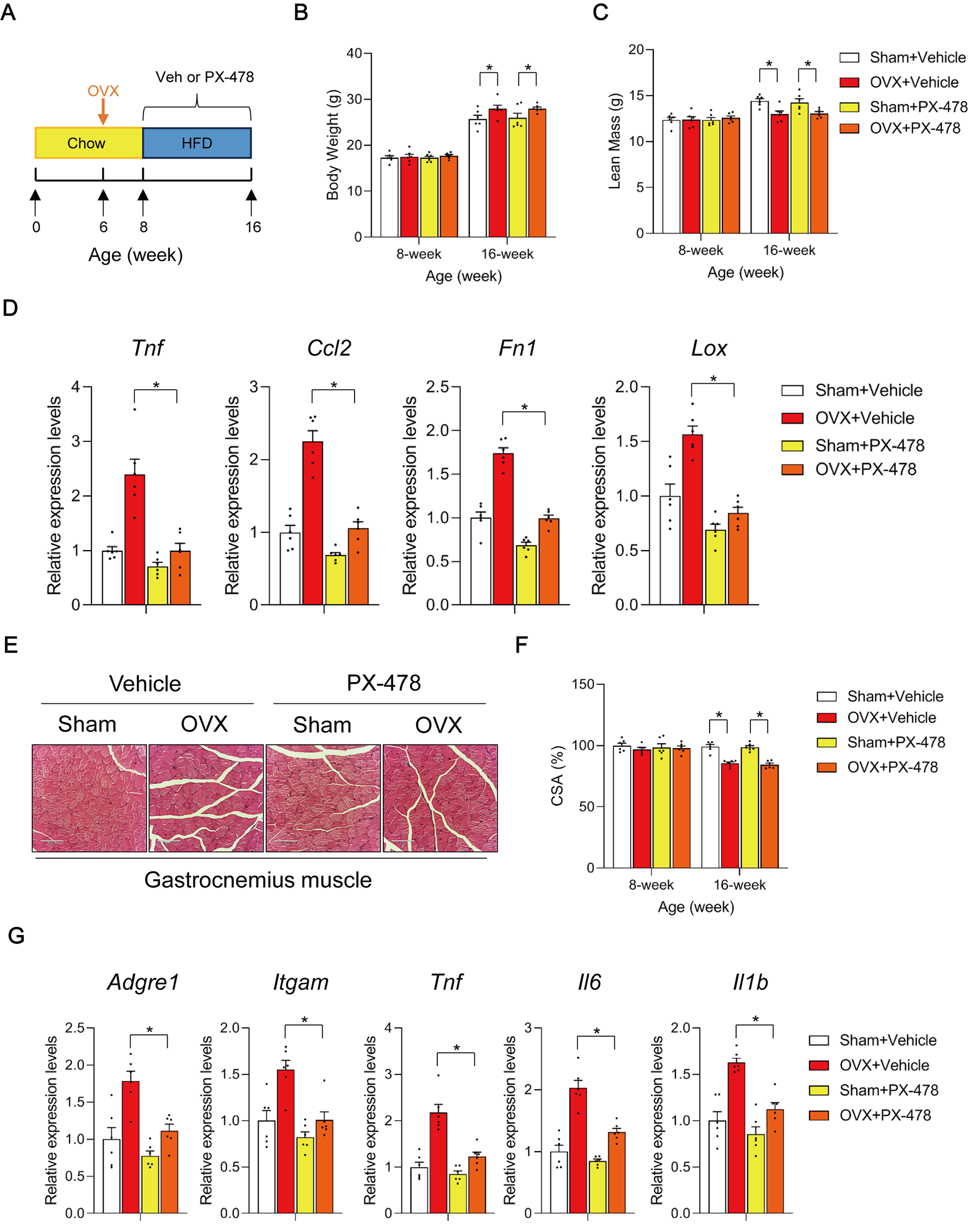

Supplement: Supplementary Figure 1 — HIF1α inhibitor improves adipose tissue remodeling and muscle inflammation in high-fat diet (HFD)-fed ovariectomized mice. (A) Schematic diagram illustrating experimental design. Six-week old wildtype C57BL/6 female mice kept on standard chow diet were operated (sham or OVX). Two weeks after the operation, sham and ovariectomized (OVX) mice were switched to HFD feeding with vehicle or PX-478 treatment for another 8 weeks. (B) Body weights of each group were measured before and after HFD feeding. n = 6 per group. Bars represent mean ± SEM. *p < 0.05 by two-way ANOVA. (C) Lean mass of each group after 8 weeks of HFD feeding. n = 6 per group. Bars represent mean ± SEM. *p < 0.05 by two-way ANOVA. (D) Relative of inflammation-related genes in rpAT from mice of the indicated groups after HFD feeding. n = 6 per group. Bars represent mean ± SEM. *p < 0.05 by two-way ANOVA. (E) Representative H&E staining of gastrocnemius muscle sections from mice of the indicated groups. Scale bar = 100 μm. (F) Cross-sectional area (CSA) of gastrocnemius muscle from mice of the indicated groups. n = 6 per group. Bars represent mean ± SEM. *p < 0.05 by two-way ANOVA. (G) Relative expression of inflammation-related genes in gastrocnemius muscle from mice of the indicated groups. n = 6 per group. Bars represent mean ± SEM. *p < 0.05 by two-way ANOVA. [file Image_1.TIF]
